# Supplementary material for: Modeling glioblastoma heterogeneity as a dynamic network of cell states
Source: Mol Syst Biol. 2021 Sep 16;17(9):e10105. doi: 10.15252/msb.202010105 (PMC8444284; doi:10.15252/msb.202010105)
Supplement: Supplementary file 5 — Source Data for Figure 3 [file MSB-17-e10105-s001.zip › Figure3A_sourcedata/GSEA_3065/nosigdb_state1.GseaPreranked.1622718409535/RICHARDS_ZHONG_NATURE_2018_ASTROCYTES_UPREG.html]

Details for gene set RICHARDS\_ZHONG\_NATURE\_2018\_ASTROCYTES\_UPREG[GSEA]

|  || Dataset | state1 |
| Phenotype | NoPhenotypeAvailable |
| Upregulated in class | na\_neg |
| GeneSet | RICHARDS\_ZHONG\_NATURE\_2018\_ASTROCYTES\_UPREG |
| Enrichment Score (ES) | -0.6298571 |
| Normalized Enrichment Score (NES) | -2.4575822 |
| Nominal p-value | 0.0 |
| FDR q-value | 0.0 |
| FWER p-Value | 0.0 |
Table: GSEA Results Summary

  

Fig 1: Enrichment plot: RICHARDS\_ZHONG\_NATURE\_2018\_ASTROCYTES\_UPREG      
 Profile of the Running ES Score & Positions of GeneSet Members on the Rank Ordered List

  

| PROBE | GENE SYMBOL | GENE\_TITLE | RANK IN GENE LIST | RANK METRIC SCORE | RUNNING ES | CORE ENRICHMENT || 1 | LGALS1 |  |  | 4 | 0.828 | 0.0164 | No |
| 2 | S100A13 |  |  | 20 | 0.627 | 0.0275 | No |
| 3 | S100A16 |  |  | 27 | 0.587 | 0.0388 | No |
| 4 | PLIN3 |  |  | 75 | 0.410 | 0.0422 | No |
| 5 | DBI |  |  | 110 | 0.352 | 0.0458 | No |
| 6 | LGALS3 |  |  | 117 | 0.348 | 0.0523 | No |
| 7 | GNG11 |  |  | 131 | 0.335 | 0.0577 | No |
| 8 | MT2A |  |  | 161 | 0.317 | 0.0611 | No |
| 9 | RGS20 |  |  | 173 | 0.309 | 0.0662 | No |
| 10 | VIM |  |  | 175 | 0.308 | 0.0724 | No |
| 11 | GNG5 |  |  | 276 | 0.269 | 0.0674 | No |
| 12 | RHOC |  |  | 316 | 0.256 | 0.0686 | No |
| 13 | JUN |  |  | 387 | 0.229 | 0.0660 | No |
| 14 | NT5E |  |  | 462 | 0.213 | 0.0626 | No |
| 15 | DCXR |  |  | 474 | 0.211 | 0.0657 | No |
| 16 | HSPB1 |  |  | 531 | 0.199 | 0.0640 | No |
| 17 | MGLL |  |  | 633 | 0.181 | 0.0572 | No |
| 18 | ANXA5 |  |  | 709 | 0.171 | 0.0528 | No |
| 19 | CNN3 |  |  | 727 | 0.168 | 0.0545 | No |
| 20 | CBR1 |  |  | 792 | 0.158 | 0.0510 | No |
| 21 | EFHD2 |  |  | 879 | 0.147 | 0.0451 | No |
| 22 | CXCL14 |  |  | 925 | 0.142 | 0.0433 | No |
| 23 | RAB34 |  |  | 947 | 0.140 | 0.0440 | No |
| 24 | CD82 |  |  | 1024 | 0.131 | 0.0388 | No |
| 25 | YIF1A |  |  | 1106 | 0.122 | 0.0328 | No |
| 26 | SPRY2 |  |  | 1171 | 0.115 | 0.0285 | No |
| 27 | C1orf122 |  |  | 1175 | 0.115 | 0.0305 | No |
| 28 | FABP5 |  |  | 1470 | 0.091 | 0.0019 | No |
| 29 | PDLIM5 |  |  | 1528 | 0.087 | -0.0022 | No |
| 30 | DHCR7 |  |  | 1553 | 0.086 | -0.0030 | No |
| 31 | SNX3 |  |  | 1585 | 0.083 | -0.0045 | No |
| 32 | CDC42EP1 |  |  | 1644 | 0.079 | -0.0089 | No |
| 33 | SLC3A2 |  |  | 1646 | 0.079 | -0.0074 | No |
| 34 | PHACTR2 |  |  | 1726 | 0.074 | -0.0141 | No |
| 35 | ADORA2B |  |  | 1891 | 0.066 | -0.0298 | No |
| 36 | ZFYVE21 |  |  | 1938 | 0.063 | -0.0333 | No |
| 37 | GLUL |  |  | 2130 | 0.054 | -0.0520 | No |
| 38 | RGCC |  |  | 2214 | 0.051 | -0.0596 | No |
| 39 | LYN |  |  | 2272 | 0.048 | -0.0645 | No |
| 40 | FAM84B |  |  | 2432 | 0.042 | -0.0802 | No |
| 41 | TIMP1 |  |  | 2497 | 0.040 | -0.0860 | No |
| 42 | LRRC17 |  |  | 2509 | 0.039 | -0.0863 | No |
| 43 | TRIM47 |  |  | 2525 | 0.039 | -0.0871 | No |
| 44 | SERPINB6 |  |  | 2574 | 0.037 | -0.0913 | No |
| 45 | OAT |  |  | 2778 | 0.031 | -0.1117 | No |
| 46 | SLC9A3R1 |  |  | 2862 | 0.029 | -0.1198 | No |
| 47 | RGMA |  |  | 3071 | 0.023 | -0.1409 | No |
| 48 | GNG12 |  |  | 3088 | 0.023 | -0.1421 | No |
| 49 | GULP1 |  |  | 3139 | 0.022 | -0.1468 | No |
| 50 | DARS |  |  | 3142 | 0.022 | -0.1466 | No |
| 51 | GLUD1 |  |  | 3290 | 0.018 | -0.1615 | No |
| 52 | EEPD1 |  |  | 3518 | 0.013 | -0.1847 | No |
| 53 | SLC25A18 |  |  | 3696 | 0.010 | -0.2029 | No |
| 54 | ETV5 |  |  | 3894 | 0.006 | -0.2232 | No |
| 55 | ARNTL |  |  | 4033 | 0.004 | -0.2374 | No |
| 56 | LITAF |  |  | 4039 | 0.003 | -0.2379 | No |
| 57 | PLCD1 |  |  | 4219 | 0.000 | -0.2564 | No |
| 58 | PSAT1 |  |  | 4318 | -0.001 | -0.2665 | No |
| 59 | TP53I3 |  |  | 4331 | -0.002 | -0.2678 | No |
| 60 | SASH1 |  |  | 4498 | -0.004 | -0.2849 | No |
| 61 | BMP7 |  |  | 4562 | -0.006 | -0.2913 | No |
| 62 | TCF7L2 |  |  | 4707 | -0.008 | -0.3061 | No |
| 63 | CRYL1 |  |  | 4799 | -0.009 | -0.3153 | No |
| 64 | PDGFRB |  |  | 5041 | -0.013 | -0.3401 | No |
| 65 | DOK5 |  |  | 5166 | -0.015 | -0.3526 | No |
| 66 | PHGDH |  |  | 5207 | -0.016 | -0.3564 | No |
| 67 | GNPTG |  |  | 5837 | -0.026 | -0.4211 | No |
| 68 | SFXN5 |  |  | 5908 | -0.028 | -0.4278 | No |
| 69 | ROBO3 |  |  | 5970 | -0.029 | -0.4336 | No |
| 70 | DTNA |  |  | 6001 | -0.029 | -0.4361 | No |
| 71 | TOR1AIP1 |  |  | 6024 | -0.030 | -0.4378 | No |
| 72 | ABCD3 |  |  | 6145 | -0.032 | -0.4496 | No |
| 73 | IDI1 |  |  | 6154 | -0.032 | -0.4498 | No |
| 74 | FERMT2 |  |  | 6579 | -0.040 | -0.4929 | No |
| 75 | ID1 |  |  | 6749 | -0.044 | -0.5095 | No |
| 76 | RASSF4 |  |  | 6781 | -0.045 | -0.5119 | No |
| 77 | PEA15 |  |  | 6830 | -0.046 | -0.5159 | No |
| 78 | CAMK2G |  |  | 6872 | -0.046 | -0.5192 | No |
| 79 | SIRPA |  |  | 6912 | -0.048 | -0.5223 | No |
| 80 | DDAH1 |  |  | 6943 | -0.049 | -0.5244 | No |
| 81 | SNX5 |  |  | 7025 | -0.051 | -0.5318 | No |
| 82 | EMC2 |  |  | 7044 | -0.051 | -0.5326 | No |
| 83 | TIMP3 |  |  | 7100 | -0.053 | -0.5373 | No |
| 84 | TECPR2 |  |  | 7111 | -0.053 | -0.5372 | No |
| 85 | LIPA |  |  | 7165 | -0.055 | -0.5416 | No |
| 86 | CYP51A1 |  |  | 7249 | -0.057 | -0.5491 | No |
| 87 | RAB31 |  |  | 7256 | -0.057 | -0.5485 | No |
| 88 | PBXIP1 |  |  | 7302 | -0.058 | -0.5520 | No |
| 89 | PAQR8 |  |  | 7316 | -0.058 | -0.5522 | No |
| 90 | ALDH6A1 |  |  | 7321 | -0.059 | -0.5514 | No |
| 91 | GJA1 |  |  | 7471 | -0.062 | -0.5656 | No |
| 92 | ELN |  |  | 7510 | -0.064 | -0.5682 | No |
| 93 | CCDC80 |  |  | 7525 | -0.064 | -0.5684 | No |
| 94 | EFEMP2 |  |  | 7651 | -0.068 | -0.5800 | No |
| 95 | RAMP1 |  |  | 7655 | -0.068 | -0.5789 | No |
| 96 | CTSL |  |  | 7676 | -0.069 | -0.5796 | No |
| 97 | PPP1R16A |  |  | 7703 | -0.070 | -0.5809 | No |
| 98 | SLC1A4 |  |  | 7745 | -0.072 | -0.5837 | No |
| 99 | MEIS1 |  |  | 7794 | -0.073 | -0.5872 | No |
| 100 | HOPX |  |  | 7850 | -0.075 | -0.5914 | No |
| 101 | DNER |  |  | 7852 | -0.075 | -0.5899 | No |
| 102 | BBS2 |  |  | 7875 | -0.076 | -0.5907 | No |
| 103 | IFI27L1 |  |  | 7968 | -0.080 | -0.5986 | No |
| 104 | SOAT1 |  |  | 8031 | -0.082 | -0.6033 | No |
| 105 | GNG7 |  |  | 8050 | -0.083 | -0.6035 | No |
| 106 | P3H4 |  |  | 8081 | -0.084 | -0.6049 | No |
| 107 | PLEC |  |  | 8086 | -0.084 | -0.6036 | No |
| 108 | ELOVL5 |  |  | 8115 | -0.086 | -0.6048 | No |
| 109 | GPR137B |  |  | 8169 | -0.089 | -0.6085 | No |
| 110 | ENHO |  |  | 8176 | -0.089 | -0.6073 | No |
| 111 | LRP4 |  |  | 8181 | -0.089 | -0.6059 | No |
| 112 | SLC38A3 |  |  | 8217 | -0.090 | -0.6078 | No |
| 113 | PDLIM3 |  |  | 8278 | -0.093 | -0.6121 | No |
| 114 | CDC42EP4 |  |  | 8286 | -0.094 | -0.6109 | No |
| 115 | CA12 |  |  | 8291 | -0.094 | -0.6094 | No |
| 116 | APOE |  |  | 8322 | -0.095 | -0.6106 | No |
| 117 | ADD3 |  |  | 8338 | -0.096 | -0.6102 | No |
| 118 | TNFRSF19 |  |  | 8355 | -0.097 | -0.6099 | No |
| 119 | ID2 |  |  | 8360 | -0.097 | -0.6083 | No |
| 120 | C3orf70 |  |  | 8375 | -0.098 | -0.6078 | No |
| 121 | PREX1 |  |  | 8406 | -0.099 | -0.6089 | No |
| 122 | SHISA4 |  |  | 8468 | -0.103 | -0.6132 | No |
| 123 | GRB14 |  |  | 8474 | -0.103 | -0.6116 | No |
| 124 | SYPL1 |  |  | 8546 | -0.108 | -0.6168 | No |
| 125 | RCN1 |  |  | 8553 | -0.108 | -0.6152 | No |
| 126 | UBL3 |  |  | 8573 | -0.110 | -0.6149 | No |
| 127 | HADHB |  |  | 8718 | -0.119 | -0.6274 | Yes |
| 128 | AIF1L |  |  | 8722 | -0.119 | -0.6253 | Yes |
| 129 | SERPINE2 |  |  | 8736 | -0.120 | -0.6243 | Yes |
| 130 | TEX264 |  |  | 8744 | -0.121 | -0.6225 | Yes |
| 131 | ZFP36L1 |  |  | 8789 | -0.124 | -0.6246 | Yes |
| 132 | P4HA1 |  |  | 8810 | -0.125 | -0.6241 | Yes |
| 133 | TRIB2 |  |  | 8853 | -0.129 | -0.6259 | Yes |
| 134 | C2orf72 |  |  | 8855 | -0.129 | -0.6233 | Yes |
| 135 | ID4 |  |  | 8856 | -0.129 | -0.6207 | Yes |
| 136 | BDH2 |  |  | 8927 | -0.136 | -0.6252 | Yes |
| 137 | PHYHIPL |  |  | 8937 | -0.137 | -0.6234 | Yes |
| 138 | MT3 |  |  | 8954 | -0.139 | -0.6222 | Yes |
| 139 | TSPAN6 |  |  | 8985 | -0.142 | -0.6225 | Yes |
| 140 | LEPROT |  |  | 8994 | -0.142 | -0.6204 | Yes |
| 141 | MAGT1 |  |  | 9028 | -0.145 | -0.6209 | Yes |
| 142 | GATM |  |  | 9049 | -0.147 | -0.6200 | Yes |
| 143 | COMT |  |  | 9058 | -0.148 | -0.6178 | Yes |
| 144 | FADS2 |  |  | 9078 | -0.150 | -0.6168 | Yes |
| 145 | KLHDC8A |  |  | 9117 | -0.153 | -0.6176 | Yes |
| 146 | CD63 |  |  | 9126 | -0.154 | -0.6153 | Yes |
| 147 | ZFP36L2 |  |  | 9162 | -0.160 | -0.6157 | Yes |
| 148 | GDPD2 |  |  | 9198 | -0.165 | -0.6160 | Yes |
| 149 | HEY1 |  |  | 9215 | -0.168 | -0.6142 | Yes |
| 150 | LIFR |  |  | 9216 | -0.168 | -0.6108 | Yes |
| 151 | AASS |  |  | 9237 | -0.171 | -0.6094 | Yes |
| 152 | LRIG1 |  |  | 9256 | -0.173 | -0.6078 | Yes |
| 153 | G6PC3 |  |  | 9273 | -0.175 | -0.6059 | Yes |
| 154 | NOTCH2 |  |  | 9279 | -0.177 | -0.6028 | Yes |
| 155 | HES1 |  |  | 9280 | -0.177 | -0.5992 | Yes |
| 156 | PDPN |  |  | 9284 | -0.177 | -0.5960 | Yes |
| 157 | ARHGEF26 |  |  | 9286 | -0.178 | -0.5925 | Yes |
| 158 | FAT1 |  |  | 9301 | -0.181 | -0.5903 | Yes |
| 159 | ABHD3 |  |  | 9348 | -0.189 | -0.5912 | Yes |
| 160 | MSMO1 |  |  | 9391 | -0.199 | -0.5915 | Yes |
| 161 | PMP22 |  |  | 9392 | -0.199 | -0.5875 | Yes |
| 162 | TFPI |  |  | 9427 | -0.206 | -0.5868 | Yes |
| 163 | HSD17B12 |  |  | 9436 | -0.208 | -0.5834 | Yes |
| 164 | ATP1B2 |  |  | 9441 | -0.210 | -0.5796 | Yes |
| 165 | SEMA6D |  |  | 9442 | -0.210 | -0.5753 | Yes |
| 166 | LGALS3BP |  |  | 9447 | -0.212 | -0.5715 | Yes |
| 167 | TM7SF2 |  |  | 9450 | -0.213 | -0.5674 | Yes |
| 168 | GPRC5B |  |  | 9454 | -0.214 | -0.5633 | Yes |
| 169 | SOX8 |  |  | 9456 | -0.215 | -0.5591 | Yes |
| 170 | PLEKHB1 |  |  | 9466 | -0.218 | -0.5556 | Yes |
| 171 | NOTCH3 |  |  | 9501 | -0.226 | -0.5545 | Yes |
| 172 | DAG1 |  |  | 9514 | -0.229 | -0.5511 | Yes |
| 173 | HTRA1 |  |  | 9515 | -0.229 | -0.5465 | Yes |
| 174 | HES4 |  |  | 9536 | -0.234 | -0.5438 | Yes |
| 175 | SPRY1 |  |  | 9540 | -0.236 | -0.5394 | Yes |
| 176 | BCAP29 |  |  | 9547 | -0.238 | -0.5352 | Yes |
| 177 | NLGN3 |  |  | 9554 | -0.241 | -0.5309 | Yes |
| 178 | DKK3 |  |  | 9558 | -0.244 | -0.5263 | Yes |
| 179 | ITPR2 |  |  | 9560 | -0.244 | -0.5214 | Yes |
| 180 | CST3 |  |  | 9581 | -0.254 | -0.5184 | Yes |
| 181 | TNC |  |  | 9605 | -0.262 | -0.5154 | Yes |
| 182 | TMEM132B |  |  | 9613 | -0.264 | -0.5108 | Yes |
| 183 | MRC2 |  |  | 9620 | -0.266 | -0.5060 | Yes |
| 184 | SEZ6L |  |  | 9628 | -0.272 | -0.5013 | Yes |
| 185 | IFITM3 |  |  | 9643 | -0.277 | -0.4971 | Yes |
| 186 | APBB2 |  |  | 9645 | -0.277 | -0.4916 | Yes |
| 187 | ABAT |  |  | 9646 | -0.278 | -0.4859 | Yes |
| 188 | ITGAV |  |  | 9650 | -0.282 | -0.4805 | Yes |
| 189 | RFX4 |  |  | 9655 | -0.285 | -0.4752 | Yes |
| 190 | SPATA6 |  |  | 9657 | -0.286 | -0.4695 | Yes |
| 191 | LRRN3 |  |  | 9659 | -0.286 | -0.4638 | Yes |
| 192 | MFGE8 |  |  | 9665 | -0.291 | -0.4584 | Yes |
| 193 | TSC22D4 |  |  | 9677 | -0.296 | -0.4536 | Yes |
| 194 | SOX2 |  |  | 9680 | -0.298 | -0.4477 | Yes |
| 195 | BCHE |  |  | 9684 | -0.300 | -0.4419 | Yes |
| 196 | SEMA6A |  |  | 9690 | -0.304 | -0.4363 | Yes |
| 197 | NTRK2 |  |  | 9692 | -0.307 | -0.4302 | Yes |
| 198 | PTTG1IP |  |  | 9699 | -0.313 | -0.4245 | Yes |
| 199 | TSPAN3 |  |  | 9713 | -0.326 | -0.4192 | Yes |
| 200 | HMGCS1 |  |  | 9718 | -0.329 | -0.4130 | Yes |
| 201 | CSPG5 |  |  | 9721 | -0.330 | -0.4065 | Yes |
| 202 | MYO10 |  |  | 9725 | -0.335 | -0.4000 | Yes |
| 203 | PLTP |  |  | 9727 | -0.336 | -0.3933 | Yes |
| 204 | MLC1 |  |  | 9728 | -0.338 | -0.3865 | Yes |
| 205 | LAMB2 |  |  | 9734 | -0.344 | -0.3800 | Yes |
| 206 | SLC4A4 |  |  | 9749 | -0.355 | -0.3743 | Yes |
| 207 | SOX9 |  |  | 9754 | -0.358 | -0.3674 | Yes |
| 208 | SDC3 |  |  | 9756 | -0.360 | -0.3602 | Yes |
| 209 | ITM2C |  |  | 9758 | -0.363 | -0.3530 | Yes |
| 210 | OLFM2 |  |  | 9760 | -0.365 | -0.3457 | Yes |
| 211 | SCD |  |  | 9762 | -0.370 | -0.3383 | Yes |
| 212 | ETV1 |  |  | 9764 | -0.371 | -0.3309 | Yes |
| 213 | B2M |  |  | 9791 | -0.408 | -0.3253 | Yes |
| 214 | CLU |  |  | 9795 | -0.418 | -0.3171 | Yes |
| 215 | SPARCL1 |  |  | 9798 | -0.423 | -0.3088 | Yes |
| 216 | QKI |  |  | 9803 | -0.430 | -0.3005 | Yes |
| 217 | WSCD1 |  |  | 9808 | -0.448 | -0.2918 | Yes |
| 218 | C1orf61 |  |  | 9813 | -0.479 | -0.2825 | Yes |
| 219 | ALCAM |  |  | 9818 | -0.493 | -0.2729 | Yes |
| 220 | S100B |  |  | 9822 | -0.507 | -0.2630 | Yes |
| 221 | SLC1A3 |  |  | 9823 | -0.509 | -0.2526 | Yes |
| 222 | EDNRB |  |  | 9824 | -0.509 | -0.2423 | Yes |
| 223 | FABP7 |  |  | 9825 | -0.513 | -0.2319 | Yes |
| 224 | DDR1 |  |  | 9829 | -0.524 | -0.2216 | Yes |
| 225 | CD9 |  |  | 9832 | -0.544 | -0.2108 | Yes |
| 226 | PON2 |  |  | 9835 | -0.554 | -0.1998 | Yes |
| 227 | ADGRG1 |  |  | 9851 | -0.654 | -0.1881 | Yes |
| 228 | FAM181B |  |  | 9854 | -0.697 | -0.1742 | Yes |
| 229 | PRCP |  |  | 9855 | -0.712 | -0.1598 | Yes |
| 230 | PTPRZ1 |  |  | 9869 | -0.918 | -0.1425 | Yes |
| 231 | TTYH1 |  |  | 9872 | -1.033 | -0.1218 | Yes |
| 232 | GPM6B |  |  | 9873 | -1.091 | -0.0997 | Yes |
| 233 | PTN |  |  | 9874 | -1.107 | -0.0772 | Yes |
| 234 | GFAP |  |  | 9877 | -1.174 | -0.0537 | Yes |
| 235 | BCAN |  |  | 9878 | -1.175 | -0.0299 | Yes |
| 236 | SCRG1 |  |  | 9879 | -1.473 | -0.0000 | Yes |
Table: GSEA details [plain text format]

  

Fig 2: RICHARDS\_ZHONG\_NATURE\_2018\_ASTROCYTES\_UPREG: Random ES distribution      
 Gene set null distribution of ES for **RICHARDS\_ZHONG\_NATURE\_2018\_ASTROCYTES\_UPREG**
